# Atrial fibrillation care in rural communities: a mixed methods study of physician and patient perspectives

**DOI:** 10.1186/s12875-019-1029-1

**Published:** 2019-10-24

**Authors:** Kathy L. Rush, Lindsay Burton, Fransien Van Der Merwe, Linda Hatt, Camille Galloway

**Affiliations:** 1 School of Nursing, University of British Columbia Okanagan, 1147 Research Road, Kelowna, BC V1V 1V7 Canada; 2 Cameron Medical Clinic, 302 Cameron St, Williams Lake, BC V2G 1S9 Canada; 3 University of British Columbia Okanagan, Psychology, 1147 Research Road, Kelowna, BC V1V 1V7 Canada

**Keywords:** Atrial fibrillation, Cardiac, Virtual care, Telehealth, Rural, Physicians

## Abstract

**Background:**

Atrial fibrillation (AF) is a serious heart arrhythmia associated with devastating outcomes such as stroke. Inequitable rural AF care may put patients at risk. Virtually delivered specialty AF care offers a viable option, but stakeholder perceptions of this option within the context of rural AF care is unknown. The study purpose was to obtain patient and primary care physician perspectives of rural AF care and virtually delivered AF care as a potential option.

**Methods:**

Using a mixed methods design, AF patients (*n* = 101) and physicians (*n* = 15) from three rural communities participated in focus groups and/or surveys. Focus group data were thematically analyzed, survey data were descriptively analyzed, and data were triangulated.

**Results:**

Findings captured patients’ and physicians’ perceptions of prioritized, needs, concerns and problems in AF management, available/unavailable services, and their ideas about virtual AF care. Patients and physicians identified eclectic problems in managing AF. Overall, patients felt ill informed about managing their AF and their most salient problems related to fatigue, exercise intolerance, weight maintenance, sleep apnea, and worry about stroke and bleeding. Physicians found treating patients with co-morbidities and cognitive decline problematic and balancing risks related to anticoagulation challenging. Patients and physicians identified education as a pressing need, which physicians lacked time and resources to meet. Despite available rural services, access to primary and cardiology care was a recurring challenge, and emergency department (ED) use highly contentious but often the only option for accessing care. Physicians’ managed AF care and varied in the referrals they made, often reserving them for complex situations to avoid patient travel. Patients and providers supported a broad approach to virtual AF care, tailored to an inclusive rural patient demographic.

**Conclusions:**

The study offered valuable physician and patient perspectives on AF care in rural communities including diverse management challenges, gaps in access to primary and specialty services that made ED an often used but contentious option. Findings point to the potential value of virtual care designed to reach patients with AF across the spectrum and geared to local contexts that preserve the vital role of primary care physicians in AF care in their communities.

## Background

Complex chronic diseases are particularly challenging to manage in rural communities with limited access to resources [1]. Atrial fibrillation (AF) is a serious, chronic condition that rapidly increases with advancing age [2] posing management challenges for both patients and providers. In urban centres, AF patients have access to specialists and targeted support services; however, in rural communities AF patients rely on GPs, who are also dealing with increasingly complex, older caseloads [1]. Current Canadian and international clinical guidelines for management of AF express no preference for specialist vs primary care delivery for patients with AF [3, 4]. Nevertheless specialist care is often necessary for patients with AF at some time during their disease trajectory.

Research suggests geographic variation in AF care with some evidence showing inequitable care for those in rural communities [5]. Evidence shows that rural older adults with AF receive primary care that does not consistently meet clinical guidelines [6] and have overall higher in-hospital mortality rates compared to their hospitalized urban counterparts [7]. Although not mandated, many rural patients may never see a cardiologist or have an echocardiogram [8]. Marked underutilization of warfarin has been reported in both urban and rural settings with no significant geographic differences in clinical event rates [9, 10]. Although the median number of International Normalized Ratio (INR) measurements was higher among patients with AF living in rural areas, their median time in therapeutic range was significantly lower compared to those in urban settings [11]. Even without rural-urban differences the stakes are much higher for those living in rural communities without the same access to resources.

Leveraging technology to optimize AF care for patients living in rural communities holds considerable promise in addressing inequities in care. Technology use with the AF population has shown positive outcomes. Guo et al. [12] found use of a mobile AF app that included clinical decision support tools, educational materials, patient involvement strategies, and structured follow-up improved knowledge, quality of life, and medication adherence and reduced depression and anxiety compared to usual care. Desteghe et al. [13] found that an online tailored education platform designed for patients with AF undergoing a direct current cardioversion or pulmonary vein isolation resulted in improved knowledge by the time of the procedure compared to patients with AF who received usual care. Telemedicine, involving transmission of point-of-care INR testing, resulted in improved time in therapeutic range (TTR), and in number of INRs and the frequency (decreased) between them [14]. This limited research has been exclusively urban-based, yet rural AF populations stand to benefit from technology that gives them more equitable access to resources.

Increasingly, AF clinics run by cardiologists, nurse practitioners, and/or pharmacists have evolved to meet gaps in access to specialist care. AF clinics have been shown to reduce AF-related hospitalizations, emergency department (ED) readmissions, and overall health care costs, at the same time improving health behaviors, quality of life for patients and guideline adherence [15–18]. Despite the benefits of AF clinics, patients from rural communities are still required to travel to urban centers for this specialty care. However, a virtually delivered AF clinic offers an innovative alternative in meeting the needs of rural adults with AF. Used in place of face-to-face visits, a virtual model would allow patients to stay in their communities and receive care similar to their urban counterparts.

A virtual AF clinic is a viable approach to care for the rural population yet no virtual AF models exist. However, virtual clinics that use new and emerging digital and communications technologies have been used in managing patients with a variety of chronic diseases - inflammatory bowel disease [19], diabetes [20], kidney disease [21], ophthalmology problems [22] - and for post-operative follow-up (e.g., orthopedic surgery). They have been found to facilitate rapid referral [21], improve communication with providers [20], improve clinical indicators [20, 23], improve self-efficacy [20], and achieve knowledge comparable to usual care [22]. Further, virtual visits for a variety of health issues decreased primary care visits and costs with patients reporting satisfaction and helpfulness of the visits [24].

Despite the growth and documented benefits of virtual health care, it is a departure from usual face-to-face care, therefore, patient and clinician use and receptivity may not automatically follow. One small-scale qualitative study reported that rural patients with AF and providers varied in their receptiveness to telehealth depending on their previous experience with this approach to care, satisfaction with the adequacy of their current rural health care, and perceived gaps in AF care [25]. In rural communities, primary care physicians are the main providers of AF care, yet little is known about their views on existing rural based AF care and the viability of virtually delivered care options. Understanding physician perspectives is important in determining the need for alternative care options and the patient types, situations, and problems most likely to benefit [26]. Patient perspectives of their AF care, including needs, priorities, and gaps, are equally important for tailoring virtual care options to stakeholder users [27]. The purpose of this study was to obtain patient and primary care physician perspectives of rural AF care and virtually delivered AF care being considered as a potential option. Specific study questions were: i) what are the priority management needs and concerns of rural patients with AF and their physicians? ii) what services are available/unavailable for patients with AF in rural communities, and iii) what are rural AF patient and provider perspectives of virtually delivered AF care.

## Methods

### Design

A modified version of AlDossary et al.’s [28] Telemedicine Planning Framework was broadly used to guide the current study. Three main concepts - needs, accessibility, and availability - were included. A mixed methods research design was used to provide a comprehensive look at the rural health care landscape specific to the AF population [29]. The quantitative component of the study included surveys (online or hard copy) while the qualitative component of the study included patient and physician focus groups.

### Sampling

Following joint ethics review and approval from the Behavioral Research Ethics Boards at the University of British Columbia Okanagan and Interior Health Authority (H16–02941), a purposeful sample of patients and physicians were invited to participate in the study. Each rural site within the health region had an identified champion (e.g., physician, rural site administrator) to assist with physician and patient recruitment. Physicians were invited through a letter of invitation circulated by the local champion. Other clinicians (e.g., nurse educator) were invited at the discretion of the local champion. Patients from each of the rural communities were recruited through their primary care physicians. Medical office assistants (MOA) from each physician practice identified all patients with AF from the electronic health records and mailed a prepared letter of invitation and permission to contact form to all identified patients with AF.

Participant inclusion criteria consisted of patients with a diagnosis of AF confirmed through an ECG and living in one of the three targeted communities. Participants were excluded if they had problems with memory and recall. Thiry-five physician practices, across three rural communities, had a total of approximately 542 patients with AF. Estimates of individual physician AF caseloads were difficult to determine as only eight physicians provided these numbers; of physicians who responded, there was a mean of 25 patients with AF per physician (range 15–50 patients per physician). In total, MOAs invited 417 patients to participate in a web or paper survey and/or a focus group in their local community. At the end of the survey patients were asked to indicate their interest in participating in a focus group and if favourable to contact the research coordinator (LB) for additional information. Family members who accompanied patients to the focus groups and were interested in participating were included with the patient’s consent.

### Data collection

Data were collected between March 2017 and June 2017. Each community’s AF health-related needs were identified using a combination of surveys (online, paper), focus groups (3 patient, 3 physician), and an individual interview (*n* = 1). Patient surveys addressed 12 content areas, including problematic areas of self-management; areas of information received, its source, and its helpfulness; access, availability, and use of services for AF care; and experiences with, and interest in virtual health care. Although survey items were pre-determined there was an open response option for all major content areas for participants to add any concerns or issues not addressed by the survey. The physician survey, consisted of five major content areas, which paralleled the patient survey content. Surveys were developed by the research team in consultation with a cardiologist, nurse practitioners with AF specialty, a rural physician, and a patient with AF. The patient with AF completed the online survey and provided written/oral feedback on missing content (e.g., alternative/complementary medical approaches), instructions and/or items that were difficult to interpret, and the time to completion. Changes were made to the survey based on patient feedback (see Additional file 1).

Patient focus groups asked patients to identify their most pressing problems and health needs regarding AF, resources available in their community, resource needs, as well as experiences and thoughts about virtual care. Physician focus groups asked physicians to identify the most important needs of AF patients, their priorities for care, challenges in treating AF patients, health services available and not available to their patients, as well as their thoughts on virtual AF care. In person focus groups were held in two communities, the third community’s physician focus group was held by videoconference and the one patient volunteer was interviewed by telephone. The same team member (KLR) facilitated all the focus groups.

### Analysis

Descriptive statistics were used to analyze the survey data. Using SPSS version 25, frequencies and percentages were generated for each variable, and chi-square analysis performed to determine relationships between categorical variables (e.g., education and desire for long distance care). Focus group data were thematically analyzed using NVivo10™ [30], a qualitative data management software program. The focus group questions guided the initial analysis. For each of these broader questions, three research team members (KLR, LB, CC) performed open coding of transcripts for units of meaning (e.g., words, phrases, or paragraphs). Subsequently they compared codes and clustered them into subthemes, related to each question. Quantitative and qualitative data were triangulated according to each research question.

## Results

Rural communities had population sizes that ranged from 3953 to 10,508 [31]. Locations from the urban-based AF clinic ranged from 129 to 284 miles. All three communities had community hospitals, laboratory services, and 12-lead ECG testing and pharmacies; none had echocardiography services. Only two communities had within town public transit routes, as well as special transit options for non-emergency medical appointments. The transit option for medical appointments provides residents with accessible transportation to larger nearby centres (this option does not provide transport to the urban-based AF clinic). The third community had public transportation between two small towns on Friday mornings and afternoons.

A total of 101 (of 417 receiving invitations) patients, a 25% response rate, completed surveys, 18 patients and two family members participated in focus groups, and one patient did a telephone interview. A summary of patient demographics and health history appears in Table 1. The length in years since diagnosis of AF ranged from less than a year to 44 years (median = 5.0 years). Thirty-one percent of patients had three or more co-morbidities. Fourteen physicians (9 males, 5 females), a 38.9% response rate, and one nurse educator participated in focus groups. They had a median age of 40.5 years (range: 29–63 years), and had been in their current practices from 1 to 36 years with a median of 10.5 years. Forty-three percent of physicians reported seeing patients with AF weekly and 29% on a daily basis.

**Table 1:** Summary of patient demographics

	Survey (*n* = 101)	Focus group (*n* = 18)
	*n* (%)	*n* (%)
Average age (SD)	71.8 (9.0)	72.2 (5.6)
Female	42 (41.6)	8 (44.4)
Education		
Less than high school	5 (5.0)	–
Partial high school	20 (19.8)	3 (16.7)
High school	23 (22.8)	6 (33.3)
Some college	18 (17.8)	1 (5.56)
College/University	25 (24.8)	7 (38.9)
Other	10 (9.9)	1 (5.56)
Income		
Less than $25,000	38 (37.6)	3 (16.7)
$25,000 - $50,000	37 (36.6)	11 (61.1)
$51,000 - $75,000	15 (14.9)	2 (11.2)
Over $75,000	8 (7.9)	–
Marital status		
Single	2 (2.0)	–
Married/Remarried	66 (65.3)	15 (83.5)
Common law	6 (5.9)	1 (5.5)
Divorced	6 (5.9)	1 (5.5)
Widowed	20 (19.8)	1 (5.5)
Separated	1 (1.0)	–
Ethnicity		
Caucasian	90 (89.1)	17 (94.5)
Aboriginal/First nations	3 (3.0)	–
African-Canadian	2 (2.0)	–
Other	5 (5.0)	1 (5.5)
Health history		
High blood pressure	51 (50.5)	10 (55.5)
Arthritis	37 (36.6)	9 (50.0)
Coronary hearth disease	17 (16.8)	3 (16.7)
Diabetes	25 (24.8)	4 (22.2)
Sleep apnea	24 (23.8)	6 (33.3)
Eye problems	22 (21.8)	4 (22.2)
Thyroid disease	14 (13.9)	5 (27.7)
COPD	11 (10.9)	2 (11.1)
Stroke	8 (7.9)	4 (22.2)
# of Comorbidities		
Zero	10 (9.9)	–
One	34 (33.7)	4 (22.2)
Two	26 (25.7)	6 (33.3)
Three	15 (14.9)	5 (27.7)
≥ Four	16 (15.9)	3 (16.7)
Median	2	2

Quantitative and qualitative findings for both patients and providers are integrated for each research question. Questions one and two relate to their perceptions of the priority needs and concerns of patients with AF and available/unavailable services, while question three relates to participants’ ideas about a virtual AF clinic.

### What are the priority management needs and concerns of rural patients with AF and their physicians?

Both patients with AF and physicians identified eclectic problems in managing AF, and the pressing need for patient education to address these management problems.

#### Eclectic problems

The management of AF posed problems for patients and physicians. All patients identified physical issues related to AF as the most problematic, followed by lifestyle, and emotional challenges. For patients, sleep apnea, decreased exercise tolerance, and fatigue, were extremely problematic physical issues while maintaining typical activity levels, and healthy weights were extremely problematic lifestyle issues (see Table 2). The most problematic emotional issue was worry about stroke and bleeding from anticoagulants. All but 18% of patients were on oral anticoagulants and found the management of lab tests and diet related to warfarin and out of town travel for weekly blood tests stressful and burdensome, “*My problem right now is controlling my INR. That’s my biggest headache” (Patient, Female).* Although rural patients had access to direct oral anticoagulants (DOACs), the cost and lack of insurance coverage for NOACs and fears there was no “no antidote” for this class of medications were expressed barriers to their utilization. Eventually losing the ability to drive long distances to get to appointments was a concern patients expressed. Focus group patients additionally emphasized the unknowns surrounding their AF status that created stress, and contributed to restricted activities. There were no significant relationships between patient demographics (e.g., income, education) and the identified problems.

**Table 2:** Problematic areas for atrial fibrillation patients

	Not at all (%)	Slightly/Moderately (%)	Very/Extremely (%)	Missing (%)
Physical				
Sleep apnea	51.5	27.7	13.9	6.9
Decreased exercise tolerance	18.8	55.4	21.8	4.0
Fatigue	15.8	62.4	20.8	1.0
Shortness of breath	23.8	54.5	17.7	4.0
Palpitations	30.7	45.5	15.8	7.9
Weakness	29.7	49.5	6.9	13.9
Dizziness	42.6	48.5	4.0	5.0
Fainting	78.2	7.9	4.0	9.9
Lifestyle				
Maintaining a healthy weight	48.5	35.6	14.9	1.0
Maintaining typical activity levels	33.7	48.5	14.9	3.0
Managing stress levels	45.5	47.5	5.0	2.0
Maintaining a healthy diet	62.4	32.7	4.0	1.0
Managing smoking	81.2	5.9	2.0	10.9
Managing alcohol levels	71.3	22.7	1.0	5.0
Emotional				
Worry about bleeding	42.6	46.5	8.9	2.0
Worry about stroke	35.6	54.5	7.9	2.0
Uncertainty	47.5	40.6	6.9	5.0
Anxiety	43.6	48.5	5.0	3.0
Fear	57.4	33.6	5.0	4.0
Depression	65.3	29.7	4.0	1.0

Physicians identified comorbidities and cognitive issues as the most challenging patient problems to manage in conjunction with AF. According to one physician, *“Dementia and AF are a terrible combination.”* Anticoagulant management, particularly warfarin monitoring and dose adjustments, was especially challenging with the added compliance (medications, INR, travel) complexities for patients with cognitive issues. Some physicians found warfarin the “biggest time factor” while others found it manageable, and beneficial for staying connected with their patients. Balancing the risks-benefits of warfarin in high-risk populations (e.g., fall risk, bleeding predispositions) was challenging for rural physicians, with some having experienced the death of patients due to bleeding from a fall.

*“I think the one challenge I have sometimes are the real complex patients, you know, that you try to decide if the risks and benefits of anticoagulation … you know like people with colitis, you know who have bleeding, or people that have previous GI bleeds or they … especially the elderly that fall.”*

#### Education: a pressing need

Patients and physicians identified education and information as the most pressing need. Whether diagnosed recently or many years ago, focus group patients described a sense of the unknown related to aspects of their AF, “*We need information. We’re just traveling in the dark all the time” (Patient, Female).* The “*terrible mystery of what is this thing [AF]*” *(Patient, Female)* and ongoing “in the moment” informational gaps perpetuated their need for education. Gaps included the meaning, normalcy, and impact of symptoms (e.g., chest pain) and treatment options (e.g., surgery for AF, medications), and activity conundrums. Nearly two-thirds of survey patients had received information on medication management (64.4%), but less than half of participants had received information on self-management (42.6%), lifestyle dos and do nots (48.5%), treatment options (46.5%), and symptom management (36.6%). Survey patients identified their family doctors as the most helpful sources of information, yet, focus group patients reported limited physician time for education, “*The doctors especially, they don’t seem to have the time to spend with you to try to educate you” (Patient, Male).*

Physicians also identified education as a priority especially for those with a new AF diagnosis. They described patient education as challenging for those whose AF was discovered incidentally, who had cognitive impairment, experienced failed treatments, or were highly symptomatic. Compounding the difficulties was physicians’ limited time for teaching. They admitted to “*doing the best that we can,*” often providing education only “*If we have extra time on our 10 min visit on top of anticoagulant time.*” Physicians also integrated progressive, episodic teaching, according to patient need:

*“You don’t know how long they’ve been in it [AF]; often elderly, co-morbidities and cognitive status either way, there is a lot of information you want to deliver to them but you know they’re going to absorb none of it so you have to do it in a way that will be useful. Have to do it in stages, handing them small amounts of information as they will only remember one thing.”*

### What services are available/unavailable for patients with AF in rural communities?

Patients and physicians described available rural services for managing AF but commonly raised challenges rural patients with AF faced in accessing both general primary and specialty (cardiology) health care. Both patients and physicians described ED use as highly contentious while physicians raised concerns about referrals and particularly follow-up.

#### General health care and ED services

The top five health care services survey patients identified as available and used included: lab services, family physicians, diagnostic services, pharmacy/pharmacist services, and local ED (Table 3). Focus group patients noted lack of access to some diagnostic “tests” in their rural communities that required travel, an anticipated concern when they were no longer able to drive. Survey patients considered specialist referrals (20%) and GP wait times (10%) as problematic, but two-thirds of participants expressed no concern with the decision to seek formal care. Focus group patients similarly described timely access to physician and specialist care quite challenging, often forcing reluctant use of the ED as their only option. Those likely to seek emergency care had a “when I’m not right I go” approach or experienced uncertainty about what was “normal,” yet did not always receive a supportive provider response, “*I had one doctor in emergency tell me; you can’t come running in here every time something goes wrong. And I was like I haven’t been here in like six months. And I’m scared, it’s my heart!” (Patient, Female).*

**Table 3:** Availability of health services

	Not Available (%)	Available & Used (%)	Available & Not Used (%)	Don’t Know (%)
Lab services	–	98.0	1.0	1.0
Diagnostic services	11.2	83.7	3.1	2.0
Chronic disease management program	18.7	3.3	7.7	70.3
Heart function clinic	36.3	2.2	2.2	59.3
Pharmacy/Pharmacist	–	77.1	16.7	6.3
Nurse practitioner	13.0	8.7	31.5	46.7
Family doctor	–	95.8	3.1	1.0
Heart specialist	44.1	26.9	4.3	24.7
Inpatient hospital services	6.3	38.5	19.8	35.4
Local emergency department	3.1	72.2	19.6	5.2
Counselling services	11.7	6.4	22.3	59.6
Cardiac rehabilitation program	26.3	4.2	5.3	64.2
Specific AF educational material	9.4	11.5	16.7	62.5
Recreational facilities	3.1	35.1	41.2	20.6
Alternative medicine	2.1	23.2	50.5	24.2

Rural physicians acknowledged that ED use was often the only option, however differed on appropriate/inappropriate uses of it. In some cases, physicians attributed ED usage to a lack of education, *“People often don’t know how long they should wait when they go into AF, what to do, how long to wait, is there something they can try.”* Physicians often encouraged their patients to go to the ED rather than not receive care and supported use of the ED as appropriate for the “patient truly having AF”, *“Just recently I had a man who had failed on 4*^*th*^ *cardioversion and he was in with feeling it again and I didn’t think it [going to the ED] was inappropriate. We’re not shocking again so let’s work on slowing things down.”* Physician reservations with ED use extended to a lack of access to medical records and GP follow up:

*“From the ED perspective, its follow-up – largely follow up with GPs in a timely fashion and then we have patients not connected to GPs. There is frustration that patients can’t follow up in the timeframe they would like to. I hear people say they called and they were given an appointment two or three weeks when a week is what they were hoping for.”*

#### Cardiology services

Both patients and physicians described limited access to cardiology services. Physicians described the hardship of patient travel for treatments or cardiology appointments, a challenge for older patients who they said “will just say no” for financial and physical reasons. Physicians reported a reluctance to have their patients travel unless absolutely necessary. Referrals to a cardiologist/electrophysiologists varied across physicians but, of physicians who reported this information (23%), the median referral rate was 50%. Physicians participating in focus groups perceived lower referral rates and expressed their tendency to manage their patients with AF with little to no specialty care referrals, *“Our patients don’t get referred to cardiology unless we’re having issues. We do our own cardioversions and do those fairly frequently. Ninety percent [of patients with AF] we manage on our own.”* To some extent, referrals reflected differing levels of experience and comfort with AF patients, from more experienced rural physicians managing their own patients, to an inexperienced rural physician who referred all AF patients because, *“I’m not always sure what expectations are of me”.*

Rural physicians described concerns when referring to specialty care, including lack of follow-up and complex patients. Physicians reported limited follow-up of patients who they had referred for specialty care, *“From a specialty standpoint I don’t see them [patients] being followed. I refer them and then they’re sent back and no long term follow up.”* Lack of follow-up was particularly troubling for physicians when their patients had experienced failed treatments, *“two ablations and they washed their hands of it, and that’s it.”* From a family practice perspective, physicians found such gaps difficult as *“the whole picture of family medicine is you don’t want patients to fall through the cracks.”* Physician concerns with referring complex patients stemmed from patients returning with too many medications and conflicting recommendations from different specialists, *“It’s been our experience that patients go to specialty clinics and they come back on seven medications and why would you do this. This person cannot manage this complicated regimen you’ve started them on.”* One rural physician with an AF diagnosis described the negativity and pessimism he personally experienced when receiving specialty AF care:

*“There is a pessimism, speaking from a patient’s perspective, about AF. When I showed up for my ablation, the first thing the nurse says to me is so, is this your first time here? I said, yes and I’m hoping it will be my last time and she says, oh no it won’t be your last time. We see you guys back all the time. This is the pessimism that you get.”*

### What are rural AF patient and provider perspectives on virtual AF care?

Patients and physicians endorsed a broad approach to virtual AF care. Patients supported a range of virtual options, including texting, videoconference, email, and telephone, particularly for health questions that needed immediate answers. While patient participants agreed that it was not practical for physicians to text them, they wanted a telehealth option that would give them timely access to health care professionals to assist in AF self-management. The few patient participants (2%) who had experience with telehealth reported positive experiences. However, half of those with no experience were extremely receptive to virtually delivered care (Table 4). Patients’ preferred a cardiologist travelling regularly to their communities, a current arrangement in one community and formerly in place in another community. Nevertheless, they conceded that using telehealth was a more efficient use of the specialists’ time, and videoconferencing the next best option if they couldn’t have in-person meetings. *“The teleconferencing would be really good for what I was going through here, six months or so ago, when I just wanted to see somebody like a specialist and talk to him.”* Nearly half of patients reported that it would be very or extremely important to include medication and bloodwork review and symptom management in virtual care delivery to help them self-manage their AF. Although lifestyle management was of lesser importance, nevertheless over a quarter of patients thought it was very/extremely important. Concerns were raised about the challenges of using unfamiliar technology and limited or poor internet connections in rural communities.

**Table 4:** Patient preferences for virtually delivered care

	Not at all (%)	Slightly/Moderately (%)	Very/Extremely (%)	Missing (%)
Desire for long distance care	9.9	39.6	47.5	5.0
Long distance symptom management	11.9	34.7	45.5	7.9
Long distance activity guidelines	14.9	36.6	38.6	9.9
Long distance weight management	24.8	35.6	26.7	12.9
Long distance stress management	24.8	35.6	29.7	9.9
Long distance medication review	12.9	28.7	50.5	7.9
Long distance review of bloodwork	14.9	27.7	47.5	9.9

Physicians were generally supportive of virtual AF clinic care, offering several suggestions for an approach that would best serve their communities and patients. They described existing clinic exemplars (e.g., virtual pacemaker clinic, diabetic clinic) for modelling the virtual AF clinic. Rural physicians preferred a clinic that was not aggressive in management particularly for those nearing end of life and with cognitive decline:

*“There comes a point when we should be taking those things away and saying you know your risk of falls and injuries is greater than your risk of having a stroke. You’ve decided that you don’t want to pursue these medications and we need to support patients as they withdraw medications.”*

Physicians advocated for a customized clinic to best serve the rural AF demographic that was inclusive of the entire spectrum of complexity and co-morbidities, *“It can’t just be the new onset healthy 58-year-old that has just failed cardioversion twice.”* From a rural physician’s standpoint, an inclusive approach would improve both clinic utilization and provide more balanced specialty recommendations, *“To be a really good virtual clinic try not to pick and choose the patients especially from a rural standpoint.”* Physicians also preferred a clinic approach that provided continuing follow up and not a one-time only service.

Physicians agreed that the virtual clinic could support educational needs, as well as provide rapid response for validation of symptomatic patients. Physicians suggested group video conferences for AF education delivery and a patient support group. They wanted the clinic to provide access to online resources and provide an option for patients to email a health care provider with questions when necessary. In addition, physicians expressed the need for a clinic to release easy guidelines for their patients and physicians to follow. Physicians emphasized the importance of a clinic follow up summary report to the patient’s GP and patients being instructed to follow up with their doctor.

## Discussion

This study offered valuable insights into identified problems and prioritized needs for rural older adults with AF, as well as gaps in services. Sleep apnea and decreased exercise tolerance, coupled with the maintenance of activity levels and healthy weights were among the most problematic AF challenges for rural patients. Recent research has shown that both the type of AF and its progression over time are determined by the number of AF risk factors (e.g., obesity, sleep apnea) thus the importance of aggressive risk factor management [32]. Furthermore, Mahajan et al. [32] recommend that patient education and risk factor management should be provided through a specialized AF clinic. A pan-Canadian survey of 14 AF clinics revealed 21% of referrals were for education [33].

Similar to other studies, warfarin management was challenging and stressful for both patients and providers [34]. Patients identified the importance of including medication and bloodwork review as part of telehealth delivery. In their meta-analysis of the effect of telehealth on anticoagulation management, Lee et al. [35] found that patients with AF who received telehealth had fewer major thromboembolic events than those receiving usual care. Point-of- care INR testing, found to enhance TTR [13], may be used to greater advantage in conjunction with telehealth for the rural AF population to enhance warfarin management. The potential for telehealth use with high risk AF patients with co-morbidities and cognitive issues, that made warfarin management complex for physicians in the current study, remains unknown. NOACs are an alternative to warfarin, however, Schwill et al. [36] found in their study of primary care patients with AF that patients on warfarin rarely switched to a NOAC, but new patients with AF were more likely to receive NOACs. The majority of patients in the current study had had AF for more than a year.

The over-riding need from both patients and providers was for education that could support AF self-management or reinforce the information rural physicians provided. Well over half of patients had not received information on self-management, including symptom self-management, lifestyle, and treatment options, three areas previously identified as representing important educational needs of AF patients [37]. Similarly, in two other studies, rural patients with AF identified self-management knowledge gaps and unmet educational needs they attributed to limited educational support from providers [25, 34]. For rural patients, the most important source of information was their GP, who lacked the time to provide foundational and ongoing patient education; a likely reflection of the more complex AF patient case loads of rural physicians [1]. Redman et al. [38] found that AF patients with important unmet informational needs may seek unproved advice from the internet. Both patients and physicians enthusiastically endorsed the value of virtually delivered education. Virtually delivered education for other chronic diseases (e.g., diabetes) has been shown to improve clinical indicators, knowledge, self-care, quality of life, and health care utilization [39].

Seventy percent of patients in the current study used the ED, heavy usage that may partly reflect educational gaps. Some rural physicians encouraged ED use while others, not overtly discouraging, found inappropriate usage. ED use is a costly option and not a suitable alternative for timely access to health care professionals, however, patients and physicians identified it as one of the few options available to patients with AF in rural communities. Physicians noted the lack of follow-up care after an ED visit, particularly for patients not attached to a primary care physician, a finding similarly reported with urban-based patients with AF being seen in the ED. [40] Internal medicine specialists, although highly qualified to provide AF care, were not available in the rural communities at the time of the study. The potential for telehealth to reduce health care utilization has been shown previously. Used with patients with chronic diseases (e.g., heart failure, chronic obstructive pulmonary disease, stroke), telemedicine reduced in-hospital admissions/re-admissions, length of hospital stay, and emergency department visits [41].

Referral rates, reported by only 23% of physicians, were highly variable. Physicians who managed their caseload of patients with AF with few specialist referrals described reserving referrals for older, high-risk, complex patients they found challenging to manage. Co-morbidities are one of several indicators of patient complexity [42]. Twenty-one percent of rural patients with AF in the current study had three or more co-morbidities with hypertension, diabetes, and sleep apnea among the four most prevalent conditions and posing risks for stroke. Evidence indicates family practice/internal medicine providers have older, sicker, higher risk patient caseloads compared to electrophysiologists [1, 43]. Evidence suggests that telemedicine may need to be adapted for sicker, frail, older patients with several chronic diseases, who may not derive maximal benefit compared to their younger, healthier counterparts [41]. Although rate and rhythm control issues were reasons for referral [44] in the current study, more prominent was the need of physicians for advice from specialists on balancing the treatment risks in the AF population. Continuing professional education about AF is essential for rural physicians to promote confidence in medical AF management.

Patients and physicians supported a virtual clinic for patients with AF in rural communities. Involving both stakeholder groups in planning telehealth support was critical particularly for non-emergent, but complex management of AF. The Canadian Cardiovascular Society guidelines for the management of AF support a multidisciplinary clinic for AF management to facilitate patient/provider education, enhance timely access to specialists, and promote best-practice guideline adherence [45]. The virtual AF clinic being planned was to include videoconference appointments, (approximately 4 times over a year) and a dedicated AF educational website, with content based on evidence-based research and best practice guidelines. Planning involved reconciling the tension between the complex patients rural physicians tended to refer and available AF clinic resources, and telehealth capabilities to replicate an in-person visit. Prioritized problems and informational gaps (e.g., symptom management, lifestyle, treatments, when to seek care) stakeholders identified were highlighted for inclusion in videoconference appointments and/or featured on the dedicated AF website. Medication review and management were to be important components of videoconference appointments.

This mixed-methods study provides a multifaceted assessment of AF care needs, gaps, and priorities from the perspective of key stakeholders representing three rural communities. Patient survey response rates were low, threatening representation and potentially limiting generalizability of the findings, however, sizeable focus groups provided in-depth elaboration of survey findings. Recruiting physicians through local champions may have introduced bias by contributing to the selection of physicians with similar views. Recruiting patients through rural physician practices did not capture patients without a physician and may have potentially under-represented the full spectrum of care gaps. A further limitation was incomplete information available from rural physicians about their AF patient caseloads to allow a more comprehensive picture of the rural AF patient population, such as prevalence of patients with new AF. Finally, cardiology and nursing support time to provide remote AF patient care may not be sustainable. However, it is anticipated that telehealth, in substituting for current face-to-face AF care, will promote more efficient use of time for both clinicians and patients.

## Conclusion

AF is a major public health concern that contributes to serious complications such as stroke. The study offered valuable physician and patient perspectives on AF care in rural communities including diverse problem management challenges, gaps in access to primary and specialty services that made ED an often used but contentious option. Specialty care has been shown to improve outcomes for the AF patient population but is limited in rural communities. Findings from this study indicated both rural patient and physician support for virtually delivered AF care to address AF management challenges. Designing virtual AF care inclusive of the broadest spectrum of rural AF patients and their physical, lifestyle, emotional, education, and management needs was considered essential. Virtual care tailored to the needs and priorities of rural patients and physicians may enhance patient self-management, facilitate rural physician referrals, and reduce ED usage in rural communities.

## Supplementary information

**Additional file 1.** Participant Survey. Health-related needs survey for patients with atrial fibrialltion covering 12 content areas, including problematic areas of self-management, areas of information received, its source, and its helpfulness; access, availability, and use of services for AF care; and experiences with, and interest in virtual health care.

## Data Availability

The datasets generated and/or analysed during the current study are not publicly available because consent was not obtained from participants for this purpose but are available from the corresponding author on reasonable request.

## Abbreviations

AF: Atrial Fibrillation
ED: Emergency Department
GP: General Practitioner
MOA: Medical Office Assistant
