# Supplementary material for: Atrial fibrillation care in rural communities: a mixed methods study of physician and patient perspectives
Source: BMC Fam Pract. 2019 Oct 24;20:144. doi: 10.1186/s12875-019-1029-1 (PMC6813979; doi:10.1186/s12875-019-1029-1)
Supplement: Supplementary file 1 — Additional file 1. Participant Survey. Health-related needs survey for patients with atrial fibrialltion covering 12 content areas, including problematic areas of self-management, areas of information received, its source, and its helpfulness; access, availability, and use of services for AF care; and experiences with, and interest in virtual health care. [file 12875_2019_1029_MOESM1_ESM.docx]

**Appendix: Patient Survey**

**Emotional Problems Related to AF**

1. **In your experience with atrial fibrillation, how problematic are each of the following?**

|  | Not at all | Slightly | Moderately | Very | Extremely |
| --- | --- | --- | --- | --- | --- |
| Anxiety |  |  |  |  |  |
| Depression |  |  |  |  |  |
| Fear |  |  |  |  |  |
| Uncertainty |  |  |  |  |  |
| Worry about stroke |  |  |  |  |  |
| Worry about bleeding due to blood thinners |  |  |  |  |  |

Other (please list and rate):

__________________________________________________________________________________________________________________________________________________________________________

**Physical Problems Related to AF**

1. **In your experience with atrial fibrillation, how problematic are each of the following?**

|  | Not at all | Slightly | Moderately | Very | Extremely |
| --- | --- | --- | --- | --- | --- |
| Fatigue/tiredness |  |  |  |  |  |
| Dizziness |  |  |  |  |  |
| Palpitations (rapid heartbeat) |  |  |  |  |  |
| Weakness |  |  |  |  |  |
| Shortness of breath |  |  |  |  |  |
| Fainting |  |  |  |  |  |
| Decreased exercise tolerance |  |  |  |  |  |
| Sleep Apnea |  |  |  |  |  |

Other (please list and rate):

________________________________________________________________________________________________________________________________________________________

**Lifestyle Challenges Related to AF**

1. **With regards to your atrial fibrillation, how problematic are each of the following?**

|  | Not at all | Slightly | Moderately | Very | Extremely |
| --- | --- | --- | --- | --- | --- |
| Your diet |  |  |  |  |  |
| Your weight |  |  |  |  |  |
| Your stress levels |  |  |  |  |  |
| Your lack of activity |  |  |  |  |  |
| Your smoking habits |  |  |  |  |  |
| Your alcohol intake |  |  |  |  |  |

Other (please list and rate):

________________________________________________________________________________________________________________________________________________________

**Receipt and Helpfulness of Information in Managing AF**

**4a. In managing your atrial fibrillation, have you received information on the following topics?**

|  | Yes | No |
| --- | --- | --- |
| Treatment options |  |  |
| Symptom management |  |  |
| Medication  management |  |  |
| Self Management |  |  |
| Lifestyle do’s and don’ts |  |  |

**4b. If so, how helpful have you found the information for each of the topics?**

|  | Not at all | Slightly | Moderately | Very | Extremely |
| --- | --- | --- | --- | --- | --- |
| Treatment options |  |  |  |  |  |
| Symptom management |  |  |  |  |  |
| Medication  management |  |  |  |  |  |
| Self Management |  |  |  |  |  |
| Lifestyle do’s and don’ts |  |  |  |  |  |

Other (please list and rate):

__________________________________________________________________________________________________________________________________________________

**Helpfulness of Sources of AF Information**

1. **How helpful do you find the following sources of information about atrial fibrillation?**

|  | Not at all | Slightly | Moderately | Very | Extremely |
| --- | --- | --- | --- | --- | --- |
| Pharmacists |  |  |  |  |  |
| Family Doctor |  |  |  |  |  |
| Cardiologist |  |  |  |  |  |
| Nurse/Nurse Practitioner |  |  |  |  |  |
| Other people with atrial fibrillation |  |  |  |  |  |
| Materials from doctors and nurses (e.g. brochure) |  |  |  |  |  |
| Books & Magazines |  |  |  |  |  |
| Online resources |  |  |  |  |  |
| YouTubes on Atrial Fibrillation |  |  |  |  |  |

Other (please list and rate):

________________________________________________________________________________________________________________________________________________________

**Accessing Services Related to Atrial Fibrillation**

1. **In your experience with atrial fibrillation, how problematic have you found the following?**

|  | Not at all | Slightly | Moderately | Very | Extremely |
| --- | --- | --- | --- | --- | --- |
| Wait times for physician appointments |  |  |  |  |  |
| Getting referrals to specialists |  |  |  |  |  |
| Making the decision to seek formal health care |  |  |  |  |  |

Other (please list and rate): ________________________________________________________________________________________________________________________________________________________

1. **Have you ever received care for your atrial fibrillation using any of the following?**

Telephone **□ Yes □ No**

Video conference **□ Yes □ No**

(talking to a Dr. or nurse using a big screen)

Email **□ Yes □ No**

Texting **□ Yes □ No**

Web Ex **□ Yes □ No**
(on line using your computer)

Pre-made videos **□ Yes □ No**

1. **If given the option, how interested would you be in long distance care for your atrial fibrillation (where you stay in your community but have contact with specialists in atrial fibrillation who live in the city)?**

|  | Not at all | Slightly | Moderately | Very | Extremely |
| --- | --- | --- | --- | --- | --- |
| Desire for long distance care |  |  |  |  |  |

1. **If you were to participate in the delivery of long distance care, how important would it be to include the following in helping you manage your atrial fibrillation?**

|  | Not at all | Slightly | Moderately | Very | Extremely |
| --- | --- | --- | --- | --- | --- |
| Symptom Management |  |  |  |  |  |
| Activity Guidelines |  |  |  |  |  |
| Weight management |  |  |  |  |  |
| Stress management |  |  |  |  |  |
| Medication review |  |  |  |  |  |
| Review of bloodwork |  |  |  |  |  |

Other (please list and rate):

________________________________________________________________________________________________________________________________________________________

1. **For each of the following services, mark the box that best describes whether it is available in your community and if you have used it.**

|  | Available  And Have Used | Available  And Have Not Used | Not Available |
| --- | --- | --- | --- |
| Lab services for bloodwork monitoring |  |  |  |
| Diagnostic services (e.g.,  electrocardiogram, echocardiogram) |  |  |  |
| A chronic disease management program |  |  |  |
| A heart function clinic |  |  |  |
| A pharmacy/pharmacist |  |  |  |
| A nurse practitioner |  |  |  |
| A family doctor |  |  |  |
| Access to heart specialists when your AF requires it |  |  |  |
| In-patient hospital services to manage your AF when needed |  |  |  |
| A local Emergency Department |  |  |  |
| Counselling services |  |  |  |
| A cardiac rehabilitation program to help with lifestyle changes |  |  |  |
| Access to educational materials specific to AF from your providers |  |  |  |
| Recreational facilities |  |  |  |

1. **Are there any resources that you would like to have access to? If so, please write down these resources in the space provided:**

__________________________________________________________________________________________________________________________________________________________________________________________________________________
